# Entomological and Molecular Surveillance of *Anopheles* Mosquitoes in Freetown, Sierra Leone, 2019

**DOI:** 10.3389/fpubh.2021.649672

**Published:** 2021-06-17

**Authors:** Ning Zhao, Ishaq Sesay, Hong Tu, Frederick Yamba, Liang Lu, Yuhong Guo, Xiuping Song, Jun Wang, Xiaobo Liu, Yujuan Yue, Haixia Wu, Qiyong Liu

**Affiliations:** ^1^State Key Laboratory of Infectious Disease Prevention and Control, Chinese Center for Disease Control and Prevention, National Institute for Communicable Disease Control and Prevention, Beijing, China; ^2^Sierra Leone-China Friendship Biosafety Laboratory, Freetown, Sierra Leone; ^3^Chinese Center for Disease Control and Prevention, National Institute of Parasitic Diseases, Shanghai, China; ^4^Ministry of Health and Sanitation of Sierra Leone, Freetown, Sierra Leone

**Keywords:** *Anopheles gambiae*, seasonal fluctuation, *plasmodium* infection rate, *kdr*, Sierra Leone

## Abstract

**Background:** Malaria is endemic in Sierra Leone, with stable and perennial transmission in all parts of the country. At present, the main prevention and control measures for mosquito vectors here involve insecticide treated nets (ITN) and indoor residual spraying (IRS). The most recent entomological surveillance was conducted prior to the civil war, between 1990 and 1994. Therefore, a new entomological surveillance required to support targeted malaria control strategies.

**Methods:** *Anopheles* mosquitoes were collected between June and December 2019 using the light trap method. On these, we conducted species identification, analyzed seasonal fluctuation and *Plasmodium* infection rate, and monitored insecticide resistance.

**Results:** Surveillance of seasonal fluctuation showed that there were two peak of *Anopheles* density in July (mean 13.67 mosquitoes/trap/night) and October (mean 13.00 mosquitoes/trap/night). Meanwhile, the lowest *Anopheles* density was seen in early September. Ninety-one representatives of *Anopheles gambiae* s.l. were selected and identified as *An. coluzzii* (*n* = 35) and *An. gambiae* s.s. (*n* = 56) using PCR. *An. coluzzii* and *An. gambiae* s.s. were found to be heterozygous resistant to the knockdown resistance (*kdr*) L1014F mutation (100%). Meanwhile, the East African mutation (*kdr* L1014S) was absent in the tested mosquitoes. Three mosquitoes that tested positive for the parasite, had an individual *Plasmodium falciparum* infection rate of 12.50, 16.67, and 14.29%. The sampling dates of positive mosquitoes were distributed in the two periods of peak *Anopheles* mosquito density.

**Conclusion:** This study identified the dominant *Anopheles* species in Freetown as *An. gambiae* while the predominant species within the *An. gambiae* complex was *An. gambiae sensu stricto*. Surveillance of seasonal fluctuations and high *P. falciparum* infection rates in *Anopheles* indicate that the alternation of drought and rainy seasons from June to July, and from October to November, are the key periods for malaria control and prevention in Freetown, Sierra Leone. The high frequency of *kdr* allele mutations in *An. gambiae* calls for close monitoring of vector susceptibility to insecticides and tracing of resistance mechanisms in order to develop more effective vector control measures and strategies.

## Introduction

Malaria remains a global public health crisis (1). According to the world malaria report 2019, an estimated 228 million cases of malaria occurred worldwide in 2018. Moreover, the African Region of the World Health Organization (WHO), which still bears the largest burden of malaria morbidity, reported 213 million cases (93%) in 2018. Globally, 272 000 (67%) malaria deaths were estimated to occur in children aged <5 years. Almost 85% of all deaths in 2018 occurred in 20 countries in the WHO African Region and India, including Sierra Leone (2). Malaria, an endemic disease in Sierra Leone, has spread steadily throughout the country. Studies have reported high parasite prevalence (3) and high mortality (4) in children <5 years of age in Sierra Leone. Although, pregnant women and children <5 are the most affected, the entire population is at risk and under the burden of malaria. Indeed, malaria accounts for 40.3% of outpatient morbidity at all ages in this country (5).

Malaria is a mosquito-borne infectious disease caused by parasitic protozoa of the genus *Plasmodium*, transmitted by female *Anopheles* mosquitoes (6). Despite the heavy burden of malaria, the control of vector-borne diseases in Sierra Leone focuses mainly on case management, while limited effort is made to reduce and interrupt transmission. Mosquito vector control is one of the most effective methods for reducing malaria transmission (7, 8). At present, the main prevention and control measures of mosquito vectors in Sierra Leone are insecticide treated nets (ITN) and indoor residual spraying (IRS). The effective implementation of these measures must be based on entomological surveillance. However, the most recent entomological surveillance (9) was conducted before the civil war of 1990–1994. Freetown, the capital of Sierra Leone, is the largest city and an economic, commercial, educational, and cultural center of this country. Accurate reporting of *Anopheles* seasonal fluctuation and parasitic infection rate in vectors, species identification, and the monitoring of insecticide resistance in Freetown need to be undertaken to support local targeted malaria control strategies.

*Anopheles gambiae* s.l. is the predominant malaria vector in Sierra Leone. Among the *An. gambiae* complex, only *An. colluzzi* (formerly M-form *An. gambiae*)*, An. gambiae* s.s. (formerly S-form *An. gambiae*), and *An. melas* were recorded in Sierra Leone (10). The main insecticides used in long-lasting insecticidal nets (LLINs) are pyrethroids, which are the only WHO-recommended type of insecticides. However, resistance of malaria vectors to pyrethroids is widespread in Africa (11) and resistance to other insecticides has been recorded in many countries (12–16). Pyrethroid insecticides work by targeting voltage-gated sodium channels. Different point mutations were found in the S6 transmembrane segment of the sodium channel gene domain II. In a wide range of insects, mutations can lead to a phenotype called knockdown resistance (*kdr*) (17–19). In *An. gambiae*, the *kdr* L1014F mutation is widely found in West and Central Africa, while the *kdr* L1014S mutation is more limited to East Africa (20). The transmission of these mutations among *An. gambiae* individuals will seriously affect malaria vector control strategies that are based on chemical insecticides (21). *Plasmodium falciparum* is the main parasite in all serious malaria cases and is further involved in more than 90% of uncomplicated malaria cases in Sierra Leone (5). There have been limited new studies (22–24) on the *Plasmodium* infection rate of the *Anopheles* mosquito vector, and especially the comparative data on the infection rate of *An. coluzzii* and *An. gambiae* s.s. in Freetown.

In this study, the entomological and molecular surveillance of *Anopheles* mosquitoes were conducted in 2019 from June to December in Freetown, Sierra Leone. Molecular identification and insecticide resistance monitoring were performed, while the seasonal fluctuation and malaria infection rate of *Anopheles* mosquitoes were determined.

## Methods and Materials

### Study Areas

**Figure 1:**
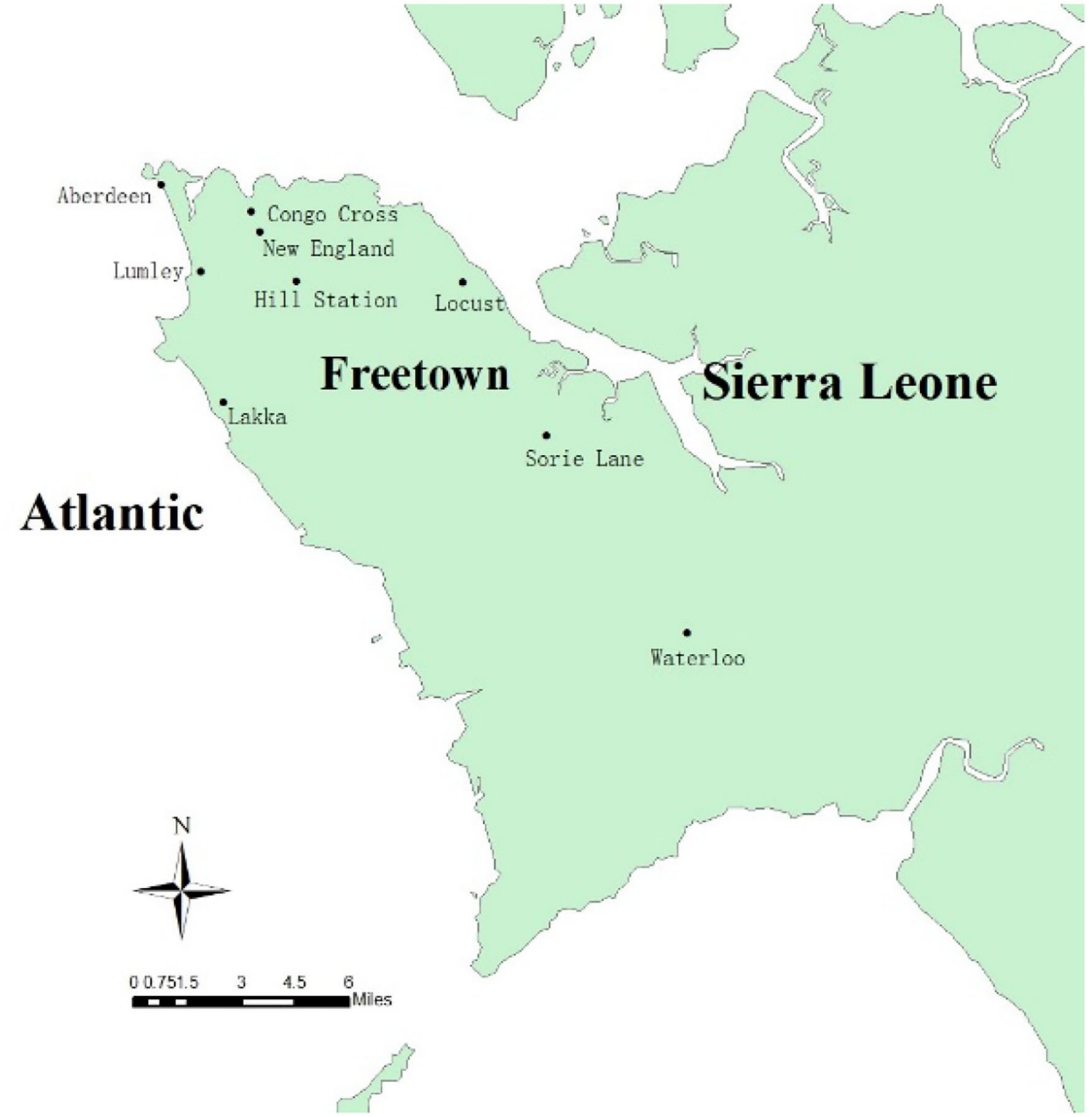
Distribution of mosquito surveillance sites in Freetown, Sierra Leone.

The current study was carried out in Freetown, the capital of Sierra Leone. Sierra Leone, located on the west coast of Africa, has a typical tropical climate with a temperature range of 21–32°C and an average temperature of 25°C. There are two main seasons in a year: the rainy season (May to October) and the dry season (November to April). Notably, there is a large amount of rainfall from July to August. The average annual rainfall is 320 cm and the relative humidity ranges from 60 to 90%. Sierra Leone has a wide range of landforms, from coastal swamps, inland swamps, and tropical rainforests, to one of the highest mountains in West Africa (Bintumani Mountain). Secondary palms are the main forms of vegetation, and they are interspersed with many marshes for rice planting. Sierra Leone has a population of about 7.8 million. The capital city, Freetown, with a population of about 1 million, is located on the Atlantic coast of the Freetown peninsula (5). The study was conducted from June to December 2019 at nine surveillance sites in Freetown. According to the geographical characteristics and whether there are local volunteers to support, nine surveillance sites were selected for monitoring, including residential areas, organization (governmental or commercial sites), hospitals, and livestock sheds. The surveillance sites were Hill Station, Sorie Lane, Aberdeen, Congo Cross, New England, Waterloo, Lakka, Lumley, and Locust sites (Table 1); of these, Lakka, Waterloo, and Sorie Lane can be found in the western area rural of Freetown, while the other six sites are in the western area urban of Freetown. The distribution of the surveillance sites is shown in Figure 1.

**Table 1:** Information of mosquito surveillance sites.

**Number**	**Surveillance site**	**Areas**	**Latitude**	**Longitude**	**Altitude (m)**	**Number of traps per time**	**Habitat**
1	Aberdeen	urban	8°29'16 “N	13°17'18” W	20	4	Residential area
2	Congo cross	urban	8°28'58 “N	13°15'26” W	40	2	Residential area
3	Hill Station	urban	8°27'14 “N	13°14'42” W	290	3	Organization
4	New England	urban	8°28'22 “N	13°14'52” W	10	2	Hospital
5	Locust	urban	8°27'57 “N	13°10'19” W	40	2	Residential area
6	Lumley	urban	8°27'25 “N	13°16'31” W	10	2	Residential area
						1	Livestock shed
7	Lakka	rural	8°24'21 “N	13°15'46” W	10	2	Residential area
						1	Livestock shed
8	Sorie lane	rural	8°23'18 “N	13°08'35” W	40	2	Hospital
						1	Residential area
						1	Livestock shed
9	Waterloo	rural	8°18'40 “N	13°4'37” W	30	4	Organization
						1	Livestock shed

### Mosquito Collection

Mosquitoes were collected using the light-trap method once a week. Approximately 2–4 mosquito traps (MYFS-HJY-1, Dongguan Houji Electronic Technology Co., Ltd.) were set at each surveillance site. These traps were set up 1 h before sunset and the collection nets were collected 1 h after sunrise the following day. Subsequently, the mosquitoes were brought back to the laboratory for morphological identification (25). The mosquitoes were monitored weekly and stored in the laboratory at −40°C for further analysis.

### DNA Extraction

Genomic DNA was extracted from individual mosquitoes using a DNA extraction kit (Bioteke, AU19014) and used for PCR analysis to identify sub-species of the *An. gambiae* complex.

### Molecular Form Detection

The molecular identification of *An. coustani* and *An. gambiae* mosquitoes, involved polymerase chain reaction (PCR) amplification and sequencing of the cytochrome oxidase subunit 1 (*COI*) region (26). *An. gambiae* individuals were identified to species using PCR (27). In performing this analysis, 25 μL of PCR mix containing UN, GA, AR, QD, and ME primers (Table 2); Premix Taq (TAKARA, RR901); water; and DNA extracted from a single mosquito was prepared. The PCR was carried out with an initial step performed at 5 min with a temperature of 94°C to activate the DNA polymerase. This was followed by 35 cycles, each comprising a 30 s denaturation at 94°C, 30 s annealing at 50°C, and 30 s extension at 72°C. The final cycle products were extended for 5 min at 72°C. Only females of *An. gambiae* (s.l.) were selected for further analysis.

**Table 2:** Sequences of the primers used throughout this study.

**Primer**	**Sequence (5^′^ to 3^′^)**
UN	GTG TGC CCC TTC CTC GAT GT
GA	CTG GTT TGG TCG GCA CGT TT
ME	TGA CCA ACC CAC TCC CTT GA
AR	AAG TGT CCT TCT CCA TCC TA
QD	CAG ACC AAG ATG GTT AGT AT
R5	GCC AAT CCG AGC TGA TAG CGC
R3	CGA ATT CTA GGG AGC TCC AG
Mop int	GCC CCT TCC TCG ATG GCA T
B/S int	ACC AAG ATG GTT CGT TGC
*kdr*-F	CAT TTT TCT TGG CCA CTG TAG TGA T
*kdr*-R	CGA TCT TGG TCC ATG TTA ATT TGC A
probe WT	VIC-CTT ACG ACT AAA TTT C-MGB
probe *kdr*W	FAM-ACG ACA AAA TTT C-MGB
probe *kdr*E	FAM-ACG ACT GAA TTT C-MGB
Agd1	ATA GAT TCC CCG ACC ATG
Agd2	AGA CAA GGA TGA TGA ACC
Agd3	AAT TTG CAT TAC TTA CGA CA
Agd4	CTG TAG TGA TAG GAA ATT TA
rPLU5	CCT GTT GTT GCC TTA AAC TTC
rPLU6	TTA AAA TTG TTG CAG TTA AAA CG
rFAL1	TTA AAC TGG TTT GGG AAA ACC AAA TAT ATT
rFAL2	ACA CAA TGA ACT CAA TCA TGA CTA CCC GTC
rVIV1	CGC TTC TAG CTT AAT CCA CAT AAC TGA TAC
rVIV2	ACT TCC AAG CCG AAG CAA AGA AAG TCC TTA
rMAL1	ATA ACA TAG TTG TAC GTT AAG AAT AAC CGC
rMAL2	AAA ATT CCC ATG CAT AAA AAA TTA TAC AAA
rOVA1	ATC TCT TTT GCT ATT TTT TAG TAT TGG AGA
rOVA2	GGA AAA GGA CAC ATT AAT TGT ATC CTA GTG

The molecular forms of *An. gambiae sensu stricto* were identified further using PCR (28). This method allows for the simultaneous identification of *An. colluzzi* and *An. gambiae* s.s. The 25 μL PCR mix contained R5, R3, Mopint, and B/Sint primers (Table 2); Premix Taq (TAKARA, RR901); water; and DNA extracted from a single mosquito. The annealing temperature for PCR amplification was 63°C.

### *kdr* Mutation Detection

Real-time polymerase chain reaction (qPCR) was used to detect *kdr* L1014F or *kdr* L1014S mutations by TaqMan analysis. (29). The primers *kdr*-Forward, *kdr*-Reverse, and the WT probe were all contained in the same reaction system. Meanwhile, the probe *kdr* W was used to detect *kdr* L1014F, and the probe *kdr* E was used to detect *kdr* L1014S (Table 2). The PCR reaction system (25 μL) contained 2 μL genomic DNA, 12.5 μL qPCR Master Mix (H&R, SJ-2101B), 900 nM of each primer, and 200 nM of each probe. The PCR was carried out using the following temperature cycling conditions: 10 min at 95°C, followed by 40 cycles at 95°C for 10 s and 60°C for 45 s.

Another PCR method was used to detect *kdr* mutation in some samples (18). Approximately 10 ± 50 ng of genomic DNA prepared as above were combined in a 25 μl total volume with the four primers Agd1, Agd2, Agd3, and Agd4 (Table 2). The PCR reaction conditions were as follows: 1 min at 94°C, 2 min at 48°C, and 2 min at 72°C for 40 cycles with a final extension step at 72°C for 10 min. Amplified fragments were analyzed using electrophoresis on a 1.5% agarose gel and were visualized through ethidium bromide staining under UV light.

### Malaria Pathogen Detection

rPLU5 and rPLU6 primers were used for the first round of amplification, and the amplified products were used as templates for the second amplification with four *Plasmodium*-specific primers. The second PCR with the rFAL1 and rFAL2 primers generated products of 205 bp for *P. falciparum*, rVIV1 and rVIV2 primers generated a 120 bp product for *Plasmodium vivax*; rMAL1 and rMAL2 primers generated a 144 bp product for *Plasmodium malariae*; and rOVA1 and rOVA2 primers generated an 800 bp product for *Plasmodium ovale* (Table 2) (30).

### Statistical Analysis

ArcGIS 10.7 was used to map the *Anopheles* mosquito surveillance sites in Freetown, Sierra Leone. Meanwhile, the Microsoft Excel 2019 software was used to analyze the monitoring data of *Anopheles* mosquitoes from the surveillance sites. This included a sum of the total number of *Anopheles* mosquitoes, and calculation of the distribution ratio and the seasonal variation of mosquito density, as well as a count of the malaria infection rate of *Anopheles* mosquitoes. SPSS v21.0 software was used for statistical analysis.

## Results

### Distribution Analysis of Collected Mosquitoes

During the period from June 26 to December 31, 2019, mosquito vector density monitoring was carried out a total of 26 times at 9 surveillance sites, and 3 012 mosquitoes were collected. Among these, 2 556 *Culex* mosquitoes accounted for 84.86%, 410 *Anopheles* mosquitoes of malaria vectors accounted for 13.61%, 43 *Aedes* mosquitoes accounted for 1.43%, and 3 other mosquito species accounted for 0.10% of the total number of mosquitoes captured. The average mosquito density in Freetown was 4.35 mosquitoes/trap/night.

The average density of *Anopheles* mosquitoes in Freetown was 0.61 mosquitoes/trap/night. The number of *Anopheles* collected at Lakka surveillance site was 330, accounting for 80.49% of the total number, and the average density of *Anopheles* was 4.78 mosquitoes/trap/night. In addition, 30 *Anopheles* were captured at the Waterloo and Locust, accounting for 7.32% of the total number, and the average density of *Anopheles* were 0.25 and 0.63 mosquitoes/trap/night. In other surveillance sites, the number of *Anopheles* trapped was lower (details are shown in Table 3). Statistics by region showed that 362 *Anopheles* were trapped in the western area rural, accounting for 88.29% of the total. The average density was 1.31 mosquitoes/trap/night. A total of 48 *Anopheles* were trapped in the western area urban, accounting for 11.71% of the total. The average density was 0.12 mosquitoes/trap/night.

**Table 3:** Trapping of *Anopheles* mosquitoes in different surveillance sites.

**Number**	**Surveillance site**	**Number of traps**	**Number of *Anopheles***	**Percentage of total (%)**	**Average density (mosquitoes/trap/night)**
1	Aberdeen	98	3	0.73	0.03
2	Congo cross	52	4	0.98	0.08
3	Hill station	78	1	0.24	0.01
4	New England	39	2	0.49	0.05
5	Lakka	69	330	80.49	4.78
6	Locust	48	30	7.32	0.63
7	Lumley	76	8	1.95	0.11
8	Sorie lane	90	2	0.49	0.02
9	Waterloo	118	30	7.32	0.25
Total		668	410	100.00	0.61

### Density of *Anopheles* Mosquitoes in Different Habitats

The results showed that the density of *Anopheles* was the highest in livestock shed (2.45 mosquitoes/trap/night). The density of *Anopheles* in residential areas was 0.60 mosquitoes/trap/night, while that in organization and hospitals was 0.11 and 0.03 mosquitoes/trap/night, respectively. The density of *Anopheles* trapped in different livestock sheds was further compared. The densities of *Anopheles* in the livestock sheds of Lakka, Waterloo, Lumley, and Sorie Lane were 8.55, 0.69, 0.12, and 0.07 mosquitoes/trap/night, respectively.

### Surveillance of Seasonal Fluctuation of *Anopheles* Density in the Lakka Community

Most of field-collected *Anopheles* mosquitoes (80.49%) were from Lakka; therefore, this study focused on the Lakka surveillance site as a representative region to conduct further research and analysis. Lakka is in the western area rural of Freetown, Sierra Leone and its environs are rice fields.

**Figure 2:**
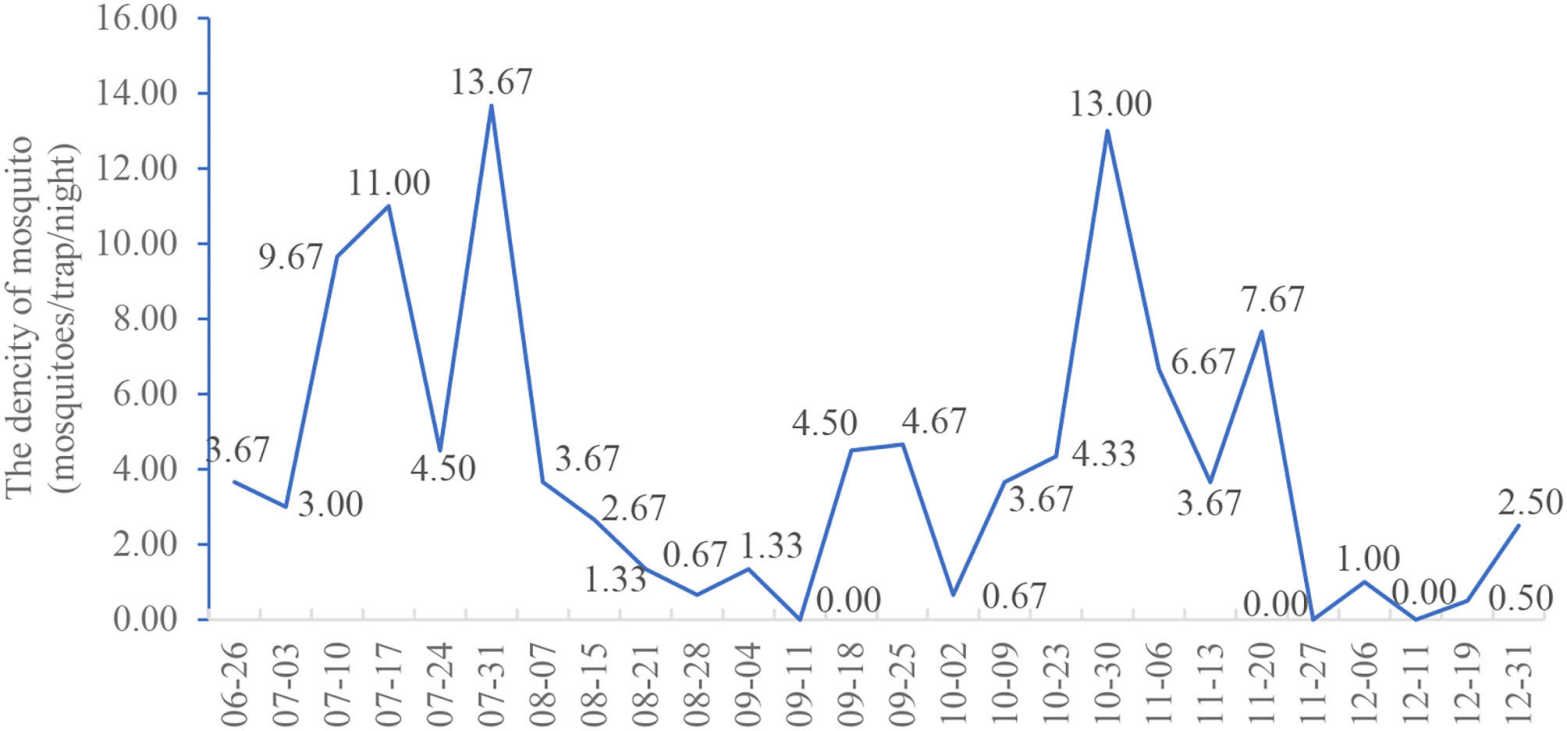
Seasonal variation of the mean density of *Anopheles* in Lakka, Freetown from June to December 2019.

The seasonal fluctuation trend of *Anopheles* density in the Lakka community is shown in Figure 2. During the monitoring period, there were two periods of peak *Anopheles* density, the first from July 3 to August 7, and the second from October 9 to November 20. The mosquito density was 13.67 mosquitoes/trap/night on July 31, while in August, the density of *Anopheles* gradually decreased reaching its lowest level in early September. The density of *Anopheles* mosquitoes then began to rise, reaching a second peak at the end of October, at 13.00 mosquitoes/trap/night. Subsequently, the *Anopheles* density again began to decrease in December.

### Molecular Identification of *Anopheles* Mosquitoes

The mosquitoes were identified morphologically and their identities were then confirmed using the *COI* sequence. From July 17, 2019 to November 6, 2019, 180 *An. gambiae* complex (85.31%) and 31 *An. coustani* (14.69%) mosquitoes were collected. The principal malaria vectors in *An. gambiae* complex are *An. gambiae sensu stricto, An. arabiensis, An. quadriannulatus* species A and *An. quadriannulatus* species B, as well as *An. melas, An. merus*, and *An. bwambae* (31). By amplifying the *IGS* gene, 91 representatives of *An. gambiae* complex were selected and identified as *An. gambiae sensu stricto* using PCR. *Anopheles gambiae sensu stricto* was recently reclassified as two species, *An. coluzzii* and *An. gambiae* s.s. Subsequently, both *An. coluzzii* and *An. gambiae* s.s. were identified (Table 4). There were 35 *An. coluzzii* mosquitoes accounting for 38.46% (35/91) and 56 *An. gambiae* s.s. mosquitoes, accounting for 61.54% (56/91) of the *An. gambiae* complex identified by molecular method.

**Table 4:** Molecular identification of members of the *An. gambiae* complex collected in the Lakka surveillance site.

**Sampling date**	**Number of samples collected**	**Number of samples tested**	***An. coluzzii***	***An. gambiae* s.s**.	**Resistance status**
2019/07/17	32	10	8	2	RS
2019/07/31	40	9	3	6	RS
2019/08/07	11	11	6	5	RS
2019/08/15	8	8	3	5	RS
2019/08/28	2	2	2	0	RS
2019/09/04	4	4	0	4	RS
2019/09/18	10	10	2	8	RS
2019/09/25	11	7	3	4	RS
2019/10/02	2	2	1	1	RS
2019/10/09	4	3	2	1	RS
2019/10/23	4	4	1	3	RS
2019/10/30	35	13	3	10	RS
2019/11/06	17	8	1	7	RS
Total	180	91	35	56	RS

*RS, heterozygous*.

### Detection of Resistance Genes

All three *kdr* alleles (*kdr* 1014L, *kdr* 1014F, and *kdr* 1014S) were detected in both *An. coluzzii* and *An. gambiae* s.s. In each *kdr* 1014F mutant assay, an intermediate increase in both VIC fluorescence and FAM fluorescence indicated a heterozygote. In each *kdr* 1014S mutant assay, a substantial increase in VIC fluorescence, with no increase in FAM fluorescence indicated that there was no *kdr* 1014S mutant. According to the above results, 35 *An. coluzzii* and 56 *An. gambiae* s.s. mosquitoes were all heterozygous resistant to the *kdr* L1014F mutation (100%) (Table 4). Some samples were confirmed by another test method. Furthermore, the East African mutation (*kdr* L1014S) was absent in the tested mosquitoes.

### Malaria Parasite Infection in Field-Collected Mosquitoes

A total of 91 *An. gambiae* complex and 31 *An. coustani* were further tested for the presence of malarial parasites. While *P. falciparum* parasites were detected in three mosquitoes, *Plasmodium vivax, Plasmodium malariae*, and *Plasmodium ovale* parasites were not found. Overall, the *An. gambiae* complex had an individual *P. falciparum* infection rate of 3.30%, while no *P. falciparum* infection was detected in the *An. coustani* individuals (Table 5). Individually, the *An. coluzzii* and *An. gambiae* s.s. mosquitoes had *P. falciparum* infection rates of 5.71 and 1.79%, respectively. *An. coluzzii* was found to have a higher individual *P. falciparum* infection rate than *An. gambiae* s.s., but the difference was not statistically significant (*P* > 0.05). Meanwhile, the three *P. falciparum* parasite-positive mosquitoes had an individual *P. falciparum* infection rate of 12.50% (July 17, 2019), 16.67% (August 7, 2019), and 14.29% (November 6, 2019), respectively. The sampling dates of positive mosquitoes were distributed in the two periods of peak *Anopheles* density (from July 3 to August 7 and from October 9 to November 20).

**Table 5:** *Plasmodium falciparum* infection of *An. gambiae* complex individuals collected in Lakka from June to December 2019.

**Sampling date**	***An. coluzzii***			***An. gambiae*** **s.s**.		
	***P. falciparum* infected**	**Number of samples tested**	**Infection rate (%)**	***P. falciparum* infected**	**Number of samples tested**	**Infection rate (%)**
2019/07/17	1	8	12.50	0	2	0
2019/07/31	0	3	0	0	6	0
2019/08/07	1	6	16.67	0	5	0
2019/08/15	0	3	0	0	5	0
2019/08/28	0	2	0	0	0	0
2019/09/04	0	0	0	0	4	0
2019/09/18	0	2	0	0	8	0
2019/09/25	0	3	0	0	4	0
2019/10/02	0	1	0	0	1	0
2019/10/09	0	2	0	0	1	0
2019/10/23	0	1	0	0	3	0
2019/10/30	0	3	0	0	10	0
2019/11/06	0	1	0	1	7	14.29
Total	2	35	5.71	1	56	1.79

## Discussion

The monitoring results showed that *Culex* accounted for the highest proportion of the total number of mosquitoes, which was the dominant species in Freetown, followed by *Anopheles*, and *Aedes*, which had the lowest density. However, *Aedes* was mainly active in the daytime, and the mosquito traps were used at night, so the data of *Aedes* could not represent the real situation of the region. In this study, 6 surveillance sites were in the western area urban, while only 3 surveillance sites were in the western area rural. However, the number of *Anopheles* trapped in western areas rural accounted for 88.29%, and the average density of *Anopheles* trapped was much higher than that in western urban areas. It may be that there are many paddy fields and swamps in the western area rural, which are breeding areas of *Anopheles*. These results suggest that the risk of malaria transmitting in western areas rural is higher than that in western areas urban in Freetown.

Two periods of peak *Anopheles* density appeared during our study, one in July and the other in October. In Sierra Leone, rainfall typically increases after May, which is the main reason for the increase in mosquito density. Consequently, the *Anopheles* density was high at the end of June and the beginning of July in the alternate period of the drought and rainy seasons. Heavy rains occurred in July and August, and the mosquito density gradually decreased from the end of July, reaching its lowest point in late August. Previous studies have shown that it takes at least seven days for mosquitoes to develop from eggs to adults in stable water and suitable temperature conditions (32). The lower adult mosquito density may be attributed to rainfall washing away mosquito eggs and larvae from breeding sites during periods of heavy rain. In other studies, it has been reported that large amounts of rain can affect mosquito reproduction (33). In addition, it was easy to form a stable water body for mosquito breeding in September, when the rainfall decreased. The increase of breeding places will lead to the increase of mosquito density (34, 35). Therefore, mosquito density began to rebound and reached its peak in October. Similarly, the observed decrease in the density of *Anopheles* may be due to a decrease in breeding sites in the dry season. Meanwhile, three *P. falciparum* parasite-positive mosquitoes were collected on July 17, August 7, and November 6, 2019, respectively, which was consistent with the peak period density of *Anopheles*. These results explain the two peaks of malaria transmission in Sierra Leone; one that begins during the rainy season in May and the second that starts toward the end of the season in October/November (36). These results suggest that the alternation of drought and rainy seasons from June to July and from October to November are the key periods for malaria control and prevention in Sierra Leone.

At the peak of the malaria epidemic, the *P. falciparum* infection rate of *An. gambiae* reached as high as 16.67%, which is similar to the infection rate in other malaria endemic areas (37–39). Three *Plasmodium*-infected *Anopheles* mosquitoes were collected during the two periods of peak *Anopheles* density and also in the malaria epidemic season. These results suggest that the high infection rate of *Anopheles* is closely related to the prevalence of malaria. It is very important to reduce the *plasmodium* infection rate of *Anopheles* for malaria control, and, therefore, case management should be strengthened. First, access to effective and timely diagnosis and treatment of malaria is a key intervention in malaria control efforts (5). However, it is very important to ensure that the patient is not bitten by mosquitoes during the illness. As a preventative measure, patients should wear long clothes and long sleeves and remove mosquito breeding places in their living environment. Finally, doctors need to strengthen publicity and education efforts for patients to enhance their public health awareness.

Historically, pyrethrum spraying began in Western Freetown to control adult mosquitoes in 1940. In 1946, the IRS was introduced in Freetown and Port Loko. The use of ITN began in 2002, mainly for pregnant women and children under 5 years old. In 2006, the nationwide free large-scale distribution of LLIN for children under 1-year-old and a measles vaccine campaign were carried out. In 2010, 2014, and 2017, the large-scale distribution of LLIN continued (5). A study in 2013 showed that high proportions of *kdr* mutations (96.2%) were detected in the *An. gambiae* populations in Freetown and the majority mosquitoes were RR homozygotes for *kdr* 1014F mutation, with very few heterozygotes (40). However, in this study, *An. coluzzii* and *An. gambiae* s.s. were all heterozygous resistant to the *kdr* L1014F mutation (100%). This phenomenon cannot be well-explained at present. It may be related to the pressure of Lakka environmental selection, or because of the limited sample size. It is worth further study in the future. This rapid decrease in susceptibility across sentinel sites may be due to the scale-up of LLINs in the country (41). Although, it has been reported that the extensive use of pyrethroids in agriculture contributes to the emergence of resistance in some parts of Africa, the large-scale increase in LLIN and IRS to control malaria is the main reason for the growing problem of resistance (42). Therefore, it is recommended that non-pyrethroid insecticides be used for IRS and LLIN to prevent and control malaria transmission, which is also suggested by WHO (43, 44). The high frequency of *kdr* allele mutation in *An. gambiae* calls for close monitoring of vector susceptibility to insecticides and tracing of resistance mechanisms in order to develop more effective vector control strategies and measures.

## Data Availability Statement

The raw data supporting the conclusions of this article will be made available by the authors, without undue reservation.

## Ethics Statement

All procedures were performed in accordance with established International Guiding Principles. The study was approved by the National Malaria Control Programme, Ministry of Health and Sanitation. Permission to collect mosquito from the fields was obtained from the field owners. Our study did not pose any danger to the communities involved or the staff that participated.

## Author Contributions

NZ, IS, HT, and FY made substantial contribution to the design of the research. NZ, IS, and HT conducted field work for mosquito collection. NZ, FY, LL, and YG had analyzed the data of mosquito surveillance. NZ, LL, XS, JW, XL, YY, and HW tested mosquitoes collected from surveillance sites. NZ, IS, LL, and FY contributed to the drafting of the manuscript and the important intellectual content herewith. QL supervised the work as the project administrator. All authors have read and agreed to the published version of the manuscript.

## Conflict of Interest

The authors declare that the research was conducted in the absence of any commercial or financial relationships that could be construed as a potential conflict of interest.
